# Giant functional parathyroid carcinoma: a case report and literature review

**DOI:** 10.3389/fonc.2023.1310290

**Published:** 2024-01-05

**Authors:** Jian Wu, Yifang Sun, Qian Zhang, Ying Lin, Pengzhen Wang, Lei Leng, Lei Cao, Feng Yu, Haiyan Deng

**Affiliations:** ^1^ Department of Otorhinolaryngology-Head and Neck Surgery, Guangzhou Red Cross Hospital of Jinan University, Guangzhou, Guangdong, China; ^2^ Department of Ophthalmology, Guangzhou Red Cross Hospital of Jinan University, Guangzhou, Guangdong, China; ^3^ Guangzhou Institute of Traumatic Surgery, Guangzhou Red Cross Hospital of Jinan University, Guangzhou, Guangdong, China; ^4^ Guangzhou Institute of Pathology, Guangzhou Red Cross Hospital of Jinan University, Guangzhou, Guangdong, China

**Keywords:** parathyroid carcinoma, diagnosis, treatment, prognosis, rare endocrine tumors

## Abstract

**Background:**

Parathyroid carcinoma is an infrequent neoplasm of the endocrine system, constituting roughly 0.5% to 5% of cases of primary hyperparathyroidism. The diagnosis of this condition presents a unique challenge for healthcare professionals.

**Case report:**

We present a case of a 77-year-old female patient who presented with a longstanding right-sided neck tumor. The Positron emission tomography-computed tomography (PET-CT) scan detected a substantial tumor situated at the inferior border of the thyroid gland. A surgical procedure was conducted, resulting in the total excision of the tumor. The diagnosis of parathyroid carcinoma was confirmed through pathological investigation. At the six-month follow-up, the patient exhibited favorable post-operative outcomes with no evidence of recurrence.

**Conclusion:**

The primary approaches for managing parathyroid carcinoma involve precise diagnosis and surgical removal. This case report provides confirmation that the implementation of rigorous treatment measures can yield a substantial improvement in the prognosis.

## Introduction

Parathyroid carcinoma is an infrequent neoplastic condition, including a minority of cases ranging from 0.5% to 5% among individuals diagnosed with primary hyperparathyroidism (PHPT) (1–3). Parathyroid carcinoma in its early stages sometimes exhibits a lack of distinct symptoms, leading to potential misdiagnosis as either thyroid neoplasms or benign parathyroid hypertrophy. According to a retrospective analysis conducted by Betea, it was shown that a mere 30% of the total 102 patients included in the study were conclusively diagnosed with parathyroid carcinoma prior to undergoing surgery (4). The utilization of assertive imaging techniques and corroborative surgical procedures is imperative for both the management and prognosis evaluation of the condition (5, 6). The present article discusses a notable instance wherein a substantial functioning parathyroid carcinoma was initially misdiagnosed as a thyroid tumor. The diagnosis was verified with preoperative examination and surgical pathology. In addition, an examination of existing literature pertaining to the diagnosis and treatment of parathyroid carcinoma is conducted in order to enhance comprehension of this particular medical issue.

## Case presentation

**Figure 1:**
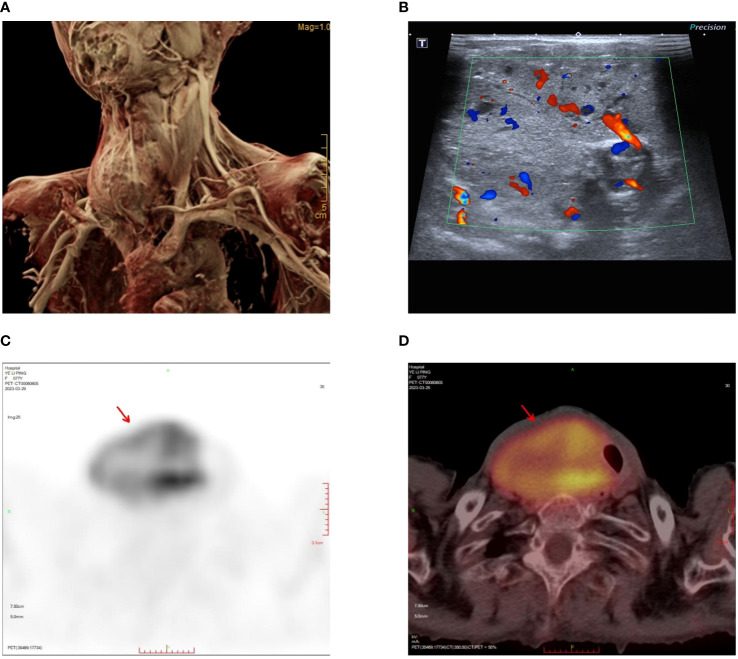
Tumor location and preoperative examinations. **(A)** MRI reconstruction showing the size and location of the tumor. **(B)** Neck ultrasound showing abundant blood supply to the tumor. **(C)** The maximum intensity projection (MIP) images summarizing the pathological distribution of [18F]-FDG in the tumor. **(D)** PET/CT scan showing heterogeneously intense FDG uptake in tumor.

The individual in question is a female patient who is 77 years of age. Two years ago, the individual fortuitously encountered an unintended discovery of a mass on the right side of her neck. Over time, the mass exhibited a progressive enlargement, although no medical intervention was pursued. In the preceding month, the lump exhibited a swift increase in size, resulting in challenges with the act of swallowing. Consequently, the individual sought medical attention at our establishment. The patient provided a negative response on the presence of symptoms such as hoarseness, breathing trouble, dizziness, or loss of strength. The physical examination identified a substantial palpable mass located on the right side of the neck. The mass had distinct boundaries, restricted movement, and did not elicit notable discomfort (**Figure 1A**).

**Figure 2:**
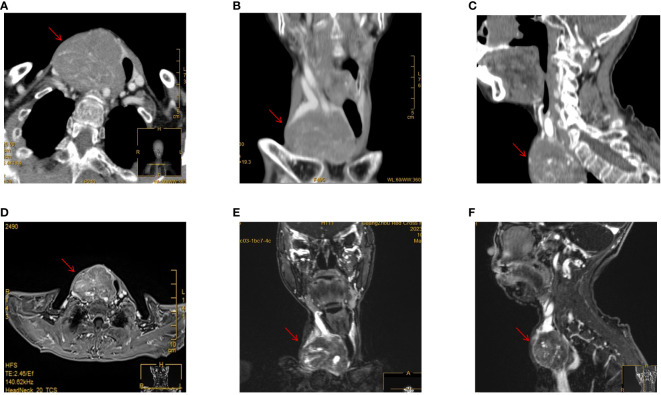
CT and MRI examinations. **(A)** Enhanced CT scan of the neck in horizontal position. **(B)** Enhanced CT scan of the neck in coronary position. **(C)** Enhanced CT scan of the neck in sagittal position. **(D)** Enhanced MRI scan of the neck in horizontal position. **(E)** Enhanced MRI scan of the neck in coronary position. **(F)** Enhanced MRI scan of the neck in sagittal position.

The neck ultrasonography examination revealed the presence of a heterogeneous hypoechoic mass measuring 14.7*5.2 cm in the inferior outer region of the right thyroid lobe. This mass exhibited distinct boundaries and contained small echogenic foci internally (**Figure 1B**). The possibility of a primary or metastatic cancer originating from the right thyroid lobe was taken into consideration. We conducted an ultrasound-guided fine-needle aspiration which indicated a benign follicular nodule, falling within the Bethesda II category. The PET-CT scan demonstrated a heterogeneously intense absorption of Fluor-18 [18F]-fluorodeoxyglucose ([18F]-FDG) in the right side of the neck, indicating a strong possibility of cancer (**Figures 1C, D**). The computed tomography (CT) scan of the neck revealed the presence of a substantial mass located on the right side. The lower boundary of the tumor extended to the upper mediastinum, and compression caused displacement of the trachea towards the left, as depicted in **Figures 2A–C**. The results of the neck Magnetic resonance imaging (MRI) revealed that the tumor is solid in nature and exhibits a plentiful blood supply. Additionally, the T1 and T2 signals of the tumor appear heterogeneous, and there is a notable enhancement observed (**Figures 2D–F**).

**Figure 3:**
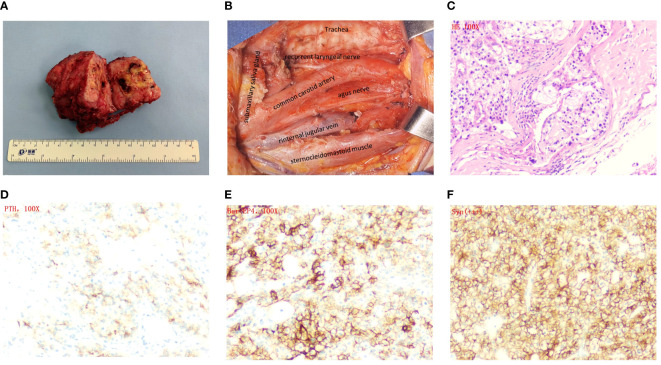
Surgery images and histopathological and immunohistochemical examinations. **(A)** Appearance of the tumor after resection. **(B)** Image of the surgical cavity after tumor resection. **(C)** Hematoxylin and eosin staining×100. **(D)** PTH×100. **(E)** Ber-EP4×100. **(F)** Syn×100.

The laboratory tests revealed that the serum calcium level was measured at 2.76 mmol/L, which exceeds the standard range of 2.1-2.6 mmol/L. Additionally, the parathyroid hormone (PTH) level was found to be 161 pg/mL, above the reference range of 14-72 pg/mL. The preliminary diagnostic prior to surgery indicated the presence of a sizable malignant neoplasm in the right lobe of the thyroid gland, accompanied by hypercalcemia. A surgical procedure was performed on the patient, which included the removal of an enlarged parathyroid tumor, lobectomy of the right thyroid, and ipsilateral cervical lymph node dissection. During the surgical procedure, it was observed that the lesion had irregular characteristics and a moderate level of adhesion. The dimensions of the lesion were measured to be 6.5×5.0×5.0 cm. Furthermore, it was found to be closely adherent to both the internal jugular vein and the common carotid artery (**Figures 3A, B**). Nevertheless, the intraoperative frozen pathology was unable to provide confirmation that it was indeed parathyroid carcinoma.

The lesion was determined to be parathyroid carcinoma based on post-operative pathology analysis (**Figure 3C**). The immunohistochemical analysis revealed robust expression of parathyroid hormone (PTH) (**Figure 3D**), as well as notable positivity for Ber-ep4 and syn (**Figures 3E, F**). The Ki-67 staining exhibited a positive rate of 5%, while the p53 staining showed a positive rate of 10% (**Supplementary Figure 1**). The patient’s parathyroid hormone (PTH) and blood calcium levels normalized after surgery, resulting in a notable alleviation of symptoms. After the operation, on the first day, the parathyroid hormone (PTH) level dropped to 1.57 pmol/L, and the calcium level was measured at 1.92 mmol/L. At the 1, 3, and 6 month follow-up visits after the operation, the parathyroid hormone (PTH) levels varied between 1.53 and 2.62 pmol/L, while the calcium levels increased to a range between 2.06 and 2.11 mmol/L. The laboratory studies and imaging did not reveal any evidence of recurrence, and the calcium levels constantly stayed within the normal range. Given these findings, it was determined that further therapy would not be pursued.

## Discussion

Parathyroid carcinoma is an infrequent malignancy of the endocrine system, and its diagnosis and treatment have consistently been subjects of significant interest within the realm of endocrinology (7, 8). It constitutes an estimated range of 0.5% to 5% of the total instances of primary hyperparathyroidism (PHPT) (9). The infrequent occurrence of this phenomenon implies that the majority of doctors are likely to come across only a minimal number of such instances throughout their professional trajectories, hence contributing to the intricacy and potential for misdiagnosis (10, 11).

The early detection of parathyroid carcinoma is a significant challenge in clinical practice. A considerable number of patients initially exhibit a lack of distinct clinical symptoms or indications, resulting in a high prevalence of misdiagnosis (12, 13).. Furthermore, the preoperative fine-needle aspiration technique presents difficulties in accurately confirming the presence of parathyroid carcinoma, resulting in a restricted rate of proper preoperative diagnosis (13, 14). The individual under examination in our study exhibited a cervical mass on the right side of their neck; nevertheless, they did not display apparent indications or physical manifestations indicative of parathyroid carcinoma in the outset. This finding aligns with other research, which has indicated that the timely detection of parathyroid carcinoma poses a significant difficulty in numerous instances.

Laboratory testing offer substantial evidence for the identification of secreting parathyroid carcinomas, as certain patients have heightened levels of blood calcium and parathyroid hormone (PTH) (15). The individual in question exhibited hypercalcemia and elevated parathyroid hormone (PTH) levels, which raised significant preoperative suspicion regarding the presence of a tumor originating from the parathyroid gland. This intervention facilitated focused preoperative preparation and strategic surgical planning. It is important to emphasize that hypercalcemia and high PTH levels alone do not definitively indicate the presence of parathyroid cancer. A comprehensive evaluation of several test results is necessary for an accurate diagnosis. Imaging modalities are crucial in the diagnostic process of parathyroid carcinoma. Computed tomography (CT) scans have the capacity to unveil significant characteristics such as tumor borders, internal necrosis, and calcification (16); MRI has the capacity to provide enhanced visualization of the extent of a tumor and its impact on adjacent tissues (17); PET-CT scans have the ability to assess the malignancy of a lesion by detecting and analyzing metabolic anomalies (15, 18). Nevertheless, relying solely on imaging is inadequate to establish a conclusive diagnosis, as it necessitates the inclusion of pathological findings. Furthermore, the utilization of imaging studies in this particular instance has provided a novel vantage point for the identification of parathyroid carcinoma, hence emphasizing the significance of imaging techniques within the diagnostic procedure.

Despite being a widely used diagnostic technique, there is still need for improvement in the accuracy of fine-needle aspiration biopsy (FNAB) for diagnosing parathyroid carcinoma (13). The gold standard for diagnosis continues to be postoperative pathological investigation (3). Common characteristics encompass invasive proliferation, cellular aberrations, tissue death, and infiltration of blood vessels (19, 20). However, these pathological alterations are not exclusive to parathyroid carcinoma, and we cannot make a diagnosis of parathyroid carcinoma solely based on these symptoms. The utilization of immunohistochemical staining that demonstrates positivity for parathyroid hormone (PTH) can serve as a conclusive method for determining the specific source of the tumor (21). A Ki-67 index exceeding 10% signifies a considerable level of cancer (22). Through a comparative analysis of published instances, a more comprehensive comprehension of the pathological attributes of this infrequent ailment and the pivotal significance of immunohistochemistry in its identification may be attained.

Currently, surgical resection remains the preferred treatment for parathyroid carcinoma (1). Numerous studies have documented the prevalent upregulation of PRAD1/cyclin D1 genes in parathyroid carcinoma, perhaps implicating their involvement in the pathogenesis of this malignancy (23). The presence or lack of genes, such as HER-2/neu, may also exhibit a correlation with the progression of diseases and the prediction of their outcomes (24, 25). Comprehensive investigations into these genetic alterations may facilitate the identification of possible treatment targets, albeit the translation of these findings into clinical practice remains remote. In this particular instance, the patient underwent a surgical resection procedure. The postoperative evaluations demonstrated successful removal of the tumor and a progressive return to normal levels of blood calcium and parathyroid hormone (PTH), providing additional evidence supporting the efficacy of the surgical resection. Nevertheless, previous research has indicated a recurrence incidence of approximately 50% following surgery, underscoring the critical importance of postoperative monitoring (14, 26). The effectiveness of medical treatment and radiation therapy in cases of metastasis or recurrence is a subject of ongoing debate within the academic community. This presents a considerable obstacle in clinical practice, highlighting the need for the exploration and implementation of novel targeted therapeutic approaches (27).

Due to the fact that it serves as a solitary case report, this study is limited in its capacity to analyze data and apply findings to a broader population. While this case offers comprehensive clinical data, its findings may not be readily applicable to all individuals with parathyroid carcinoma due to the absence of a more extensive case comparison.

## Conclusion

This case report describes an uncommon instance of parathyroid carcinoma, which manifested predominantly as hypercalcemia and elevated PTH levels. The case illustrates the significance of comprehensive preoperative assessment and preparation when determining the ultimate diagnosis and treatment plan. Although surgical resection has been demonstrated to be the most effective treatment at present, metastatic or recurrent cases still require more effective treatment strategies. In general, this case offers valuable insights into the identification and comprehension of this uncommon illness, as well as the development of efficient management and treatment strategies.

## Data availability statement

The raw data supporting the conclusions of this article will be made available by the authors, without undue reservation.

## Ethics statement

The studies involving humans were approved by Ethics Committee of Guangzhou Red Cross Hospital. The studies were conducted in accordance with the local legislation and institutional requirements. The participants provided their written informed consent to participate in this study. Written informed consent was obtained from the individual(s) for the publication of any potentially identifiable images or data included in this article.

## Author contributions

JW: Writing – original draft. YS: Writing – original draft. QZ: Writing – original draft. YL: Writing – original draft. PW: Writing – original draft. LL: Writing – original draft. LC: Writing – review & editing. FY: Writing – review & editing. HD: Writing – review & editing.

## Supplementary material

Supplementary Figure 1 Immunohistochemical examinations **(A)** Ki-67×100. **(B)** P53(wt)×100. **(C)** TG×100. **(D)** CD56×100. **(E)** CK-pan×100. **(F)** CD34×100.
