# Supplementary figures and images for: Giant functional parathyroid carcinoma: a case report and literature review
Source: Front Oncol. 2024 Jan 5;13:1310290. doi: 10.3389/fonc.2023.1310290 (PMC10797129; doi:10.3389/fonc.2023.1310290)

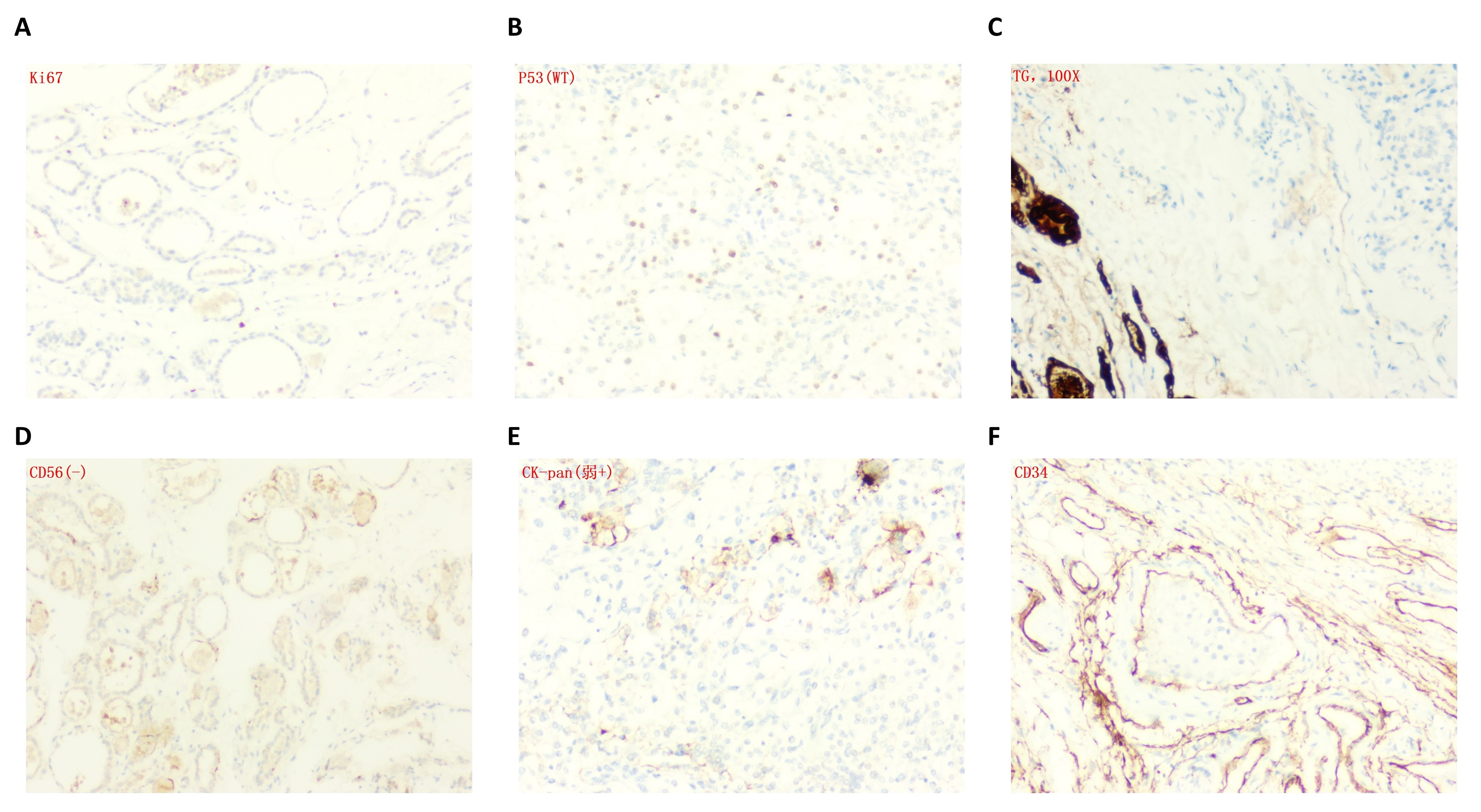

Supplement: Supplementary Figure 1 — Immunohistochemical examinations (A) Ki-67×100. (B) P53(wt)×100. (C) TG×100. (D) CD56×100. (E) CK-pan×100. (F) CD34×100. [file Image_1.jpeg]
